# Web-Based Weight Loss Intervention for Men With Type 2 Diabetes: Pilot Randomized Controlled Trial

**DOI:** 10.2196/diabetes.7430

**Published:** 2017-07-07

**Authors:** Anna Haste, Ashley J Adamson, Elaine McColl, Vera Araujo-Soares, Ruth Bell

**Affiliations:** 1 Institute of Health and Society Faculty of Medical Sciences Newcastle University Newcastle upon Tyne United Kingdom; 2 Human Nutrition Research Centre Newcastle University Newcastle upon Tyne United Kingdom; 3 Fuse–UKCRC Centre for Translational Research in Public Health Newcastle University Newcastle upon Tyne United Kingdom

**Keywords:** weight loss, Web-based intervention, feasibility studies, pilot RCT, randomized controlled trial, pilot projects, type 2 diabetes, diabetes mellitus, type 2, men, men's health, process evaluation

## Abstract

**Background:**

Rising obesity levels remain a major public health concern due to the clear link with several comorbidities such as diabetes. Diabetes now affects 6% of the UK population. Modest weight loss of 5% to 10% has been shown to be associated with significant reductions in blood sugar, lipid, and blood pressure levels. Men have been shown to be attracted to programs that do not require extensive face-to-face time commitments, illustrating the potential audience available for health behavior change via the Web.

**Objective:**

The objective of our study was to evaluate the feasibility and acceptability of a Web-based weight loss intervention in men with type 2 diabetes.

**Methods:**

We conducted a pilot, parallel 2-arm, individually randomized controlled trial with embedded process evaluation. Participants were randomly assigned in a one-to-one ratio to the usual care group or the 12-month Web-based weight loss intervention, including dietitian and exercise expert feedback. Face-to-face recruitment and assessment were performed by the researcher unblinded. Data collected included weight, height, body mass index (BMI), and waist circumference, together with an audit trail of eligibility, recruitment, retention, and adherence rates. A process evaluation (website use data and qualitative interviews) monitored adherence, acceptability, and feasibility of the intervention.

**Results:**

General practice database searches achieved the recruitment target (n=61) for the population of men with type 2 diabetes, of whom 66% (40/61) completed 3-month follow-up measurements. By 12 months, the retention rate was 52% (32/61), with 12 of the 33 men allocated to the intervention group still active on the website. The intervention was seen as acceptable by the majority of participants. We gained insights about acceptability and use of the website from the parallel process evaluation.

**Conclusions:**

Recruitment to the Web-based weight loss intervention was successful. Results are descriptive, but there were positive indications of increased weight loss (in kilograms and as a percentage), and reduced waist circumference and BMI for the intervention group from 3 to 12 months, in comparison with control. This research adds to the evidence base in relation to incorporating a Web-based weight loss intervention within the UK National Health Service (NHS). NHS weight loss services are struggling to provide sufficient referrals. Therefore, alternative modes of delivery, with the potential to reduce health professional input and time per patient while still enabling individual and tailored care, need to be investigated to identify whether they can be effective and thus benefit the NHS.

**Trial Registration:**

International Standard Randomized Controlled Trial Number (ISRCTN): 48086713; http://www.isrctn.com/ISRCTN48086713 (Archived by WebCite at http://www.webcitation.org/6rO4xSlhI)

## Introduction

The direct cost of obesity in the United Kingdom is estimated at more than £5 billion per year [1] to the National Health Service (NHS). The prevalence of obese adults (body mass index [BMI] ≥30 kg/m^2^) in England is 24.9% for men and 25.2% for women [2-4]. This is cause for concern, due to the clear link between obesity and several comorbidities such as diabetes [5,6]. The risk of developing type 2 diabetes is increased considerably for people categorized as obese compared with those who have a healthy weight [7,8], with 65% to 80% of new cases of diabetes being attributed to patients being overweight or obese [9].

Diabetes now affects 6% of the UK population, with around 90% of those having a diagnosis of diabetes found to have type 2 diabetes. Complications of diabetes affect the eyes (retinopathy), heart (cardiovascular disease), kidneys (nephropathy), and nerves and feet (neuropathy) [10]. Modest weight loss of 5% to 10% is associated with significant reductions in blood sugar, lipid, and blood pressure levels [11,12].

Recruiting men to weight loss programs is notoriously difficult, with men less likely to attend NHS or commercially run weight loss services [13-17]. Men were attracted to programs that did not require extensive face-to-face time commitments [17], suggesting the potential for men to favor or at least be accepting of Web-based interventions. A previously used individualized Web-based service was shown to be successful in decreasing glycated hemoglobin and 2-hour postprandial blood glucose test in obese patients with type 2 diabetes [18].

Evidence suggests that traditional primary care management (one-to-one dietitian or practice nurse consultations) can be costly and subject to high attrition rates [19,20]. Therefore, alternative methods for effective weight loss need to be investigated.

Using the Web for a weight loss intervention may provide a suitable alternative owing to the available audience already using the Internet. In 2016, 89% of households in Great Britain (23.7 million) had Internet access, an increase from 86% in 2015 and 57% in 2006, with 82% of adults accessing the Internet almost every day in 2016 [21]. The number of households in the United Kingdom with Web access is increasing annually in all age groups [22]. Over half (51%) of Web users actively used the Internet to investigate health issues, increasing from 18% in 2007 [21].

Internet-based interventions have the potential to minimize the stigma that may be experienced during face-to-face consultations, increase accessibility, privacy, and control for the user, and reduce the cost of an intervention [23,24].

Although the number of studies on Web-based weight loss interventions has increased, conclusions on their effectiveness still remain uncertain. Previous reviews have identified the potential of Web-based weight loss interventions to result in greater weight loss and engagement with physical activity and diet in comparison with a control group [25-27]. Intervention characteristics have been shown to be heterogeneous [26,28]. In a previous review, commonly incorporated active ingredients in Web-based weight loss interventions, identified using the Coventry, Aberdeen, and London-Refined (CALO-RE) taxonomy [29], included providing feedback on performance, planning social support or social change, prompting self-monitoring of behavior or behavioral outcome, and goal setting (behavior and outcome) [30]. The review also identified that incorporating personalized feedback within Web-based weight loss interventions led to greater weight loss in comparison with control groups providing no personalized feedback [30].

There is a range of modes of delivery that can be used when providing a Web-based weight loss intervention: websites or mobile app-based technology; automated or human feedback; and text messages, email, or Web-based messaging [30].

Web-based weight loss interventions have the potential to offer long-term programs at a low cost due to their potentially greater reach, in comparison with traditional face-to-face approaches [23,31]. Effectiveness remains unclear, and there are many uncertainties regarding feasibility and acceptability of the intervention and of trial processes. Therefore, a definitive trial, preceded by a randomized pilot trial, is needed.

The aim of this study was to evaluate the feasibility and acceptability of a Web-based weight loss intervention, and the trialing of that intervention, for men with type 2 diabetes.

## Methods

We conducted a parallel-group 2-arm patient randomized rehearsal pilot randomized controlled trial (RCT) with embedded process evaluation. The pilot RCT was multicentered, consisting of patients registered with general practices within the catchment area of County Durham and Darlington in northeast England.

The aim was to recruit and randomly allocate 60 patients. A suggested sample size for pilot trials is 30 participants per arm, to enable estimation of parameters for a future trial [32,33].

### Inclusion and Exclusion Criteria

We aimed to recruit men who had a diagnosis of type 2 diabetes and had a BMI of at least 30 kg/m^2^ but less than 40 kg/m^2^ at baseline measurement. The BMI inclusion criterion was 30 kg/m^2^ or greater, as this is the inclusion criterion for the majority of NHS tier 2 (lifestyle interventions) or tier 3 (specialist services) weight management services in England [34]. As this study was examining the change in service delivery of weight management within the NHS, we followed the criterion used within the NHS. When a patient reaches a BMI of 40 kg/m^2^, lifestyle modification may no longer be appropriate and bariatric surgery may be recommended [35]. Men had to be aged 18 years or older, with no upper age restrictions.

Participants were required to have access to the Web (at home, the workplace, or a public location) on any device (desktop computer, laptop, tablet, or mobile phone).

We excluded patients unable to give written informed consent or access the intervention in English (resource constraints precluded adaptation of the intervention for non-English speakers) or who were identified by their general practitioners (GPs) as having a contraindication to the weight loss intervention (such as previous eating disorders or other mental health problems).

### Ethics

The study was accepted onto the UK National Institute for Health Research clinical research network portfolio and registered (October 26, 2012) on a clinical trial registry (ISRCTN: 48086713). We gained NHS ethical favorable opinion from National Research Ethics Service Committee East of England - Cambridge Central Proportionate Review Sub-committee on August 9, 2012 (Research Ethics Committee reference: 12/EE/0361).

### Recruitment

We recruited participants through GP database searches; we identified participating practices through the UK Primary Care Research Network. In response to participant invitation letters, potential participants could state their intention by completing an attached reply slip and returning by reply-paid mail to the research team. Participants could also contact the research team directly via email or telephone. Those who did not want to take part in the research could return the slip and (optionally) provide a reason why. During baseline appointments in GP offices, participants provided written informed consent to the researcher (AH) prior to baseline measurements.

The researcher (AH) then randomly allocated each participant to 1 of the 2 arms using the Sealed Envelope Web-based system (Sealed Envelope Ltd). Randomization was by a one-to one allocation, to either usual care (control group) or the Web-based intervention group. We used stratification to ensure that the potential confounding variable of diabetes medication was balanced between the intervention and control arms, since this might affect outcomes. The strata were diet only, oral hypoglycemic agents, or insulin. Participants who were taking insulin and tablets were assigned to the insulin stratum. Participants were informed of allocation via postal letter by the researcher (AH). Blinding of intervention allocation was not possible for anyone involved in the pilot trial.

The control arm experienced usual care for weight loss, according to their general practice’s normal processes. This was a pragmatic trial and we did not seek to influence what was offered to the patient, with no specific arrangements to review or refer participants.

Participants randomly allocated to the intervention group were sent log-in details and encouraged to log in to the intervention website [36] before their initial face-to-face consultation with their assigned dietitian.

We asked participants randomly allocated to the intervention to state whether they were engaged in any other weight loss services. None of the intervention group were using any other services.

### Intervention Description

The website (My Dietitian) was created by PraksisCare (Odense, Denmark) based on a previous study that had identified successful weight loss via a Web-based intervention [37]. We worked together with PraksisCare to develop and adapt the My Dietitian intervention to make it relevant for use within the United Kingdom and the NHS. Table 1 describes the intervention based on the Template for Intervention Description and Replication checklist [38].

A key feature of the 12-month intervention was the Web-delivered consultations (embedded email-style messages sent within the website), which were delivered to participants by health care professionals (dietitians and exercise experts). Consultations were delivered in accordance with a scheduled protocol created prior to the start of the study (Table 1). The initial one-off face-to-face meeting with the dietitian was conducted in an hour-long appointment slot. Dietitians were expected to provide Web-based consultations on a maximum weekly basis for the first 3 months (n=12) and then monthly for the last 9 months (n=9; total planned dietitian contact n=21). Exercise experts provided Web-based consultations on a maximum monthly basis for the first 3 months (n=3) and then every 3 months for the last 9 months (n=3; total planned exercise expert contact n=6). There were thus 15 planned consultations (diet and activity) in total over the first 3 months and 27 by the end of the 12-month intervention.

Time taken to write the consultations varied across the participants based on the required advice. The content of the consultations was at the professional discretion of the dietitians and exercise experts. Every intervention participant received personalized Web-based consultations from their designated dietitian and exercise expert. This feedback was based on participant input on the website and was typically concerned with areas of improvement in relation to dietary intake and physical activity.

**Table 1:** Template for Intervention Description and Replication (TIDieR) checklist^a^ for the My Dietitian Web-based weight loss intervention.

TIDieR checklist item		Description
**What**		
	Consultant feedback	The health care professionals received training on setting SMART^b^ goals with the participants and putting together action and coping plans, addressing barrier identification, and problem solving. An initial one-off consultation with the dietitian face-to-face was then followed by a structure of scheduled Web-based consultations, with the patient also able to contact the professional in between if needed. The user received a notification that feedback was available for them to read. Consultations provided the user with information in relation to their weight status and recommendations on how to improve their behaviors. Example food diaries provided users with instructions on how to perform the behavior. (BCT^c^: provide feedback on performance; provide instruction on how to perform the behavior; provide information on consequences of behavior in general; provide information on consequences of behavior to the individual; action planning; relapse prevention and coping planning; barrier identification and problem solving; goal setting: behavior and outcome).
	Daily food intake input	Type and amount of food and time consumed. Information could be converted into calories consumed and represented in a pie chart showing percentages for food types consumed. (BCT: prompt self-monitoring of behavior; provide feedback on performance).
	Physical activity input	Type, time, and intensity of any completed physical activity, which could be translated into calories burned. (BCT: prompt self-monitoring of behavior; provide feedback on performance).
	Diet budget	Daily outline of calories consumed, calories burned and the allowance they have remaining. (BCT: Prompt self-monitoring of behavior; Provide feedback on performance).
	Body measurements	Participants had the option to record waist and weight measurements and amount of steps taken presented in a graph to display participant’s progress as part of the intervention. (BCT: prompt self-monitoring of behavior and behavioral outcomes; provide feedback on performance).
	My community	Users could interact through forums, diaries, and chat rooms. Recipes and relevant articles were available to users. (BCT: plan social support and social change).
Who provided		Registered dietitians and exercise experts (Health Improvement Specialists)
How		Individually delivered via the Web
Where		A one-off face-to-face meeting with the dietitian in the participants’ homes. Then solely Web-based delivery.
When and how much		12-month intervention. The initial one-off face-to-face meeting with the dietitian was conducted in an hour-long appointment slot. Dietitians provided Web-based consultations on a maximum weekly basis for the first 3 months (n=12) and then monthly for the last 9 months (n=9, total maximum planned dietitian contact n=21). Exercise experts provided Web-based consultations on a monthly basis for the first 3 months (n=3) and then every 3 months for the last 9 months (n=3, total planned maximum exercise expert contact n=6). Total maximum consultations over the first 3 months n=15, total maximum consultations at the end of the 12-month intervention n=27. The content of the consultations was at the professional discretion of dietitians and exercise experts.
Tailoring		Every intervention participant received personalized Web-based consultations from their designated dietitian and exercise expert. This feedback was based on participant input on the website.
Modifications		No modifications were made during the study
**Fidelity**		
	Planned	A protocol of Web-based consultation provision was created for the dietitians and exercise experts. Fidelity was assessed by monitoring website use and consultation provision by dietitians and exercise experts.
	Actual	Website use data identified the number of delivered consultations in comparison with the number planned before the start of the intervention
Included BCTs from CALO-RE taxonomy^d^		Prompt self-monitoring of behavior Prompt self-monitoring of behavioral outcomes Provide instruction on how to perform the behavior Provide information on consequences of behavior in general Provide information on consequences of behavior to the individual Provide feedback on performance Action planning Relapse prevention and coping planning Barrier identification and problem solving Goal setting (behavior) Goal setting (outcome) Planning social support and social change

^a^Based on Hoffmann et al [38].

^b^SMART: specific, measurable, agreed upon, realistic, and time-based goals.

^c^BCT: behavior change technique.

^d^CALO-RE: Coventry, Aberdeen, and London-Refined taxonomy [29].

**Figure 1:**
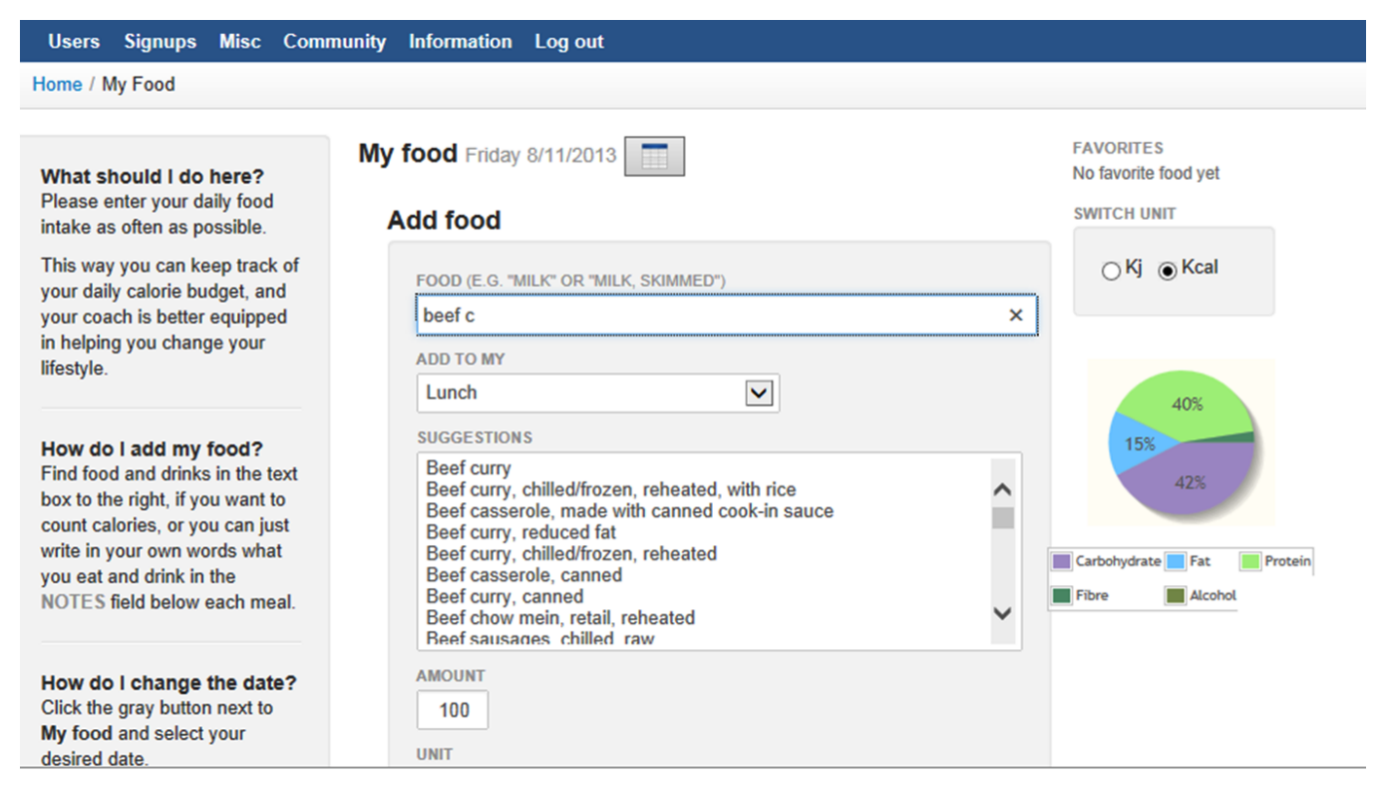
My Dietitian website screenshot of dietary intake entry page.

Website pages allowed participants to record their personal daily dietary intake (Figure 1), physical activity, or weight status, which was viewed by the health care professionals. Participants were advised to enter their dietary intake (all meals, snacks, and drinks) and physical activity on a daily basis. Using the website features was important so that the health care professionals were able to provide thorough consultations to the participants. Other features were less interactive but were provided to inform or encourage participants, such as a database of recipes, relevant articles on physical activity, diet and weight loss advice, and the ability to chat online with other participants.

We recruited and trained 2 NHS dietitians and 2 exercise experts to work on the study intervention, the My Dietitian website. The health care professionals provided quality assurance checks on the content of the website. Training consisted of 2 half-day sessions covering behavior change techniques relevant to weight loss, an overview of the Web-based intervention, and practical sessions to enable the health care professionals to become familiar with and competent at using the website.

### Primary Outcomes

The primary outcomes were recruitment and retention in the trial, as measured by rates of eligibility, response to invitation, ineligibility, declines, consent, and retention for data collection at 3 and 12 months. Within the parallel process evaluation, we examined adherence to the intervention through collection of website use data. We examined acceptability of the intervention by conducting semistructured interviews with participants.

### Secondary Outcomes

Secondary outcomes were comprehensiveness and feasibility of the measures proposed as primary or secondary outcomes in the future definitive RCT (anthropometric measures: body weight, height, BMI, and waist circumference). Losing 5% of one’s initial body weight is a target recommended by UK National Institute for Health and Care Excellence guidelines to improve health [35]. Another secondary outcome was parameter estimates of the proposed primary and secondary outcomes measures for the future definitive RCT to inform sample size calculations.

Reporting of the rehearsal pilot RCT follows the Consolidated Standards of Reporting Trials (CONSORT) statement extension to randomized pilot and feasibility trials guidelines [39] (see Multimedia Appendix 1 for the study’s CONSORT checklist [40]).

### Data Collection

Data collection points were at baseline, 3 months, and 12 months and were completed in GP offices. Rates of eligibility, recruitment, randomization, retention, attrition, and adherence were logged from the initial invitation letters through intervention allocation (baseline) to follow-up (3 and 12 months), enabling an audit trail to be maintained [41].

We collected process evaluation data for adherence by tracking and monitoring website use. Website data were collected in relation to number of website log-ins for participants, self-monitoring diaries completed (food and dietary intake and exercise entries), number of consultations made by the health professionals to the participants, and Web-based messages sent to the health professionals by the participants. Any food and dietary intake that was entered on the website on a given day was classified as 1 entry, and the same also applied to exercise entries.

We examined acceptability and feasibility through semistructured interviews conducted once with participants, lasting between 15 and 60 minutes, at the end of the 12-month study.

Anthropometric measures were collected at baseline, 3 months, and 12 months. We recorded height using a Leicester height stadiometer (Marsden Weighing Group Ltd, Rotterham, England), with participants asked to stand as straight as possible with their shoes off. Body weight was measured using calibrated Shekel personal floor scales (H151-7, Class III; Shekel Scales Ltd, Lower Galilee, Israel), allowing capacity weight up to 250 kg. Each participant was required to produce 2 body weight readings to check for consistency (within 0.1 kg). If these were not consistent then a third reading was required, and the average of the 3 readings was used as the final body weight recording. Participants remained clothed but were asked to remove coats and shoes. The Shekel scales also allowed a participant’s height to be entered along with body weight to calculate a BMI recording. Waist circumference was measured, midway between the lowest rib and the iliac crest, underneath clothing, using a tape measure.

### Data Analysis

We examined the number of participants and percentages to identify rates of eligibility, response to invitation, ineligibility, declines, consent, and retention at baseline, 3 months, and 12 months.

The recorded website data were examined to identify average number of log-ins for participants, self-monitoring diaries completed (dietary intake and exercise inputs), consultations by health professionals to the participants in comparison with scheduled consultations, and diaries and messages sent to the consultants by participants. We also examined adherence to the intervention in terms of the number of users and nonusers of the website at each time point to identify adherence over time and by population group.

Interviews were recorded and transcribed. Qualitative data were imported into NVivo 10 software (QSR International) and analyzed using framework analysis, a 5-step process: familiarization, identification of a thematic framework, indexing, charting, and mapping (interpretation) [42,43].

We used descriptive statistics to characterize rates of completion for anthropometric measures, rates of implausible values, and 5-figure summaries (minimum, maximum, median, and lower and upper quartiles). We also calculated means and standard deviations to inform sample size calculations for a potential definitive trial. Quantitative data were analyzed using IBM SPSS Statistics version 21.0 software (IBM Corporation).

## Results

### Eligibility, Recruitment, Retention, and Attrition

A total of 8 general practices agreed to perform database searches to identify potential participants, but 1 did not complete the searches due to time pressures. Practice size ranged from 1663 to 19,976 patients and varied in terms of location: town centers (n=3), housing estates (n=2), and rural villages (n=2).

**Figure 2:**
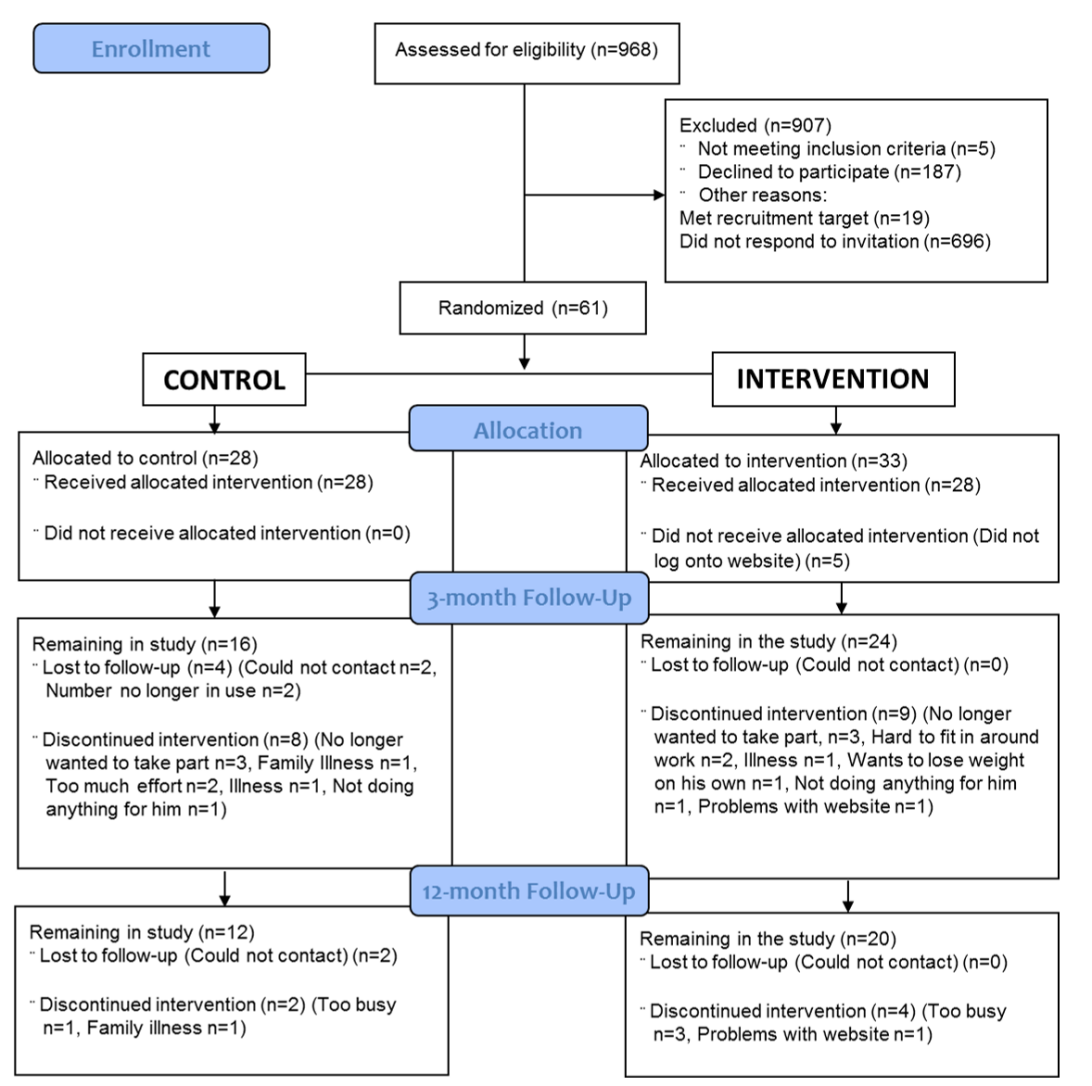
Consolidated Standards of Reporting Trials (CONSORT) recruitment flow diagram.

Figure 2 shows the CONSORT diagram. GP database searches achieved the recruitment target after a period of 5 months, with a total of 61 men providing consent and being randomly allocated. The first patient was recruited during November 2012 and March 2013, with the last 12-month follow-up appointment held in March 2014. Of the participants, 66% (40/61) completed 3-month follow-up measurements. By 12 months, retention rates had 32/61 (52%) of the men remaining.

### Response Rates

Men with diabetes (n=968) were identified from database searches (Figure 2). The response rate, expressed as those who stated an interest in joining the study as a percentage of those contacted by their practice, was 85 of 968 (8.8%). Only a small proportion of these were found to be ineligible at the baseline appointment due to changes in BMI (5/968, 0.5%).

Of those invited to the study, 187 of 968 (19.3%) of the men with diabetes explicitly declined to the invitation letter, and 115 of 187 (61.5%) did not give a reason for nonparticipation. The most common reasons for declining participation were no Web access (29/187, 15.5%), work commitments (12/187, 6.4%), poor health (10/187, 5.4%), and age (8/187, 4.3%). Those who declined due to lack of Web access did so in response to the invitation letter. Although this meant they were not eligible for participation, as these patients declined the invitation letter before any contact with the research team, they were never seen at the baseline assessment and therefore were not formally excluded. The vast majority of invited participants did not respond to the letter (696/968, 71.9%).

Data completion rates and retention rates decreased over the study time period (baseline to 3 months to 12 months). For the control group, 12 of 28 (43%) participants had dropped out by 3 months, and 16 of 28 (57%) had dropped out by the end of the study (12 months). For the intervention group, attrition was lower, with 9 of 33 (27%) participants dropping out by 3 months and 13 of 33 (39%) dropping out by 12 months. The main reasons for participants leaving the study were being too busy or having family or work commitments, for both the intervention- and the control-arm participants (Figure 2).

### Anthropometric Measures

Table 2 shows baseline demographic and anthropometric descriptive statistics.

Change variables relate to change in measurements from baseline to 3 months and baseline to 12 months (Table 3). All changes, at both 3 and 12 months, were decreases (ie, positive changes). Of the remaining men in the intervention, 8 lost 5% of their initial body weight at 12 months in comparison with 4 of the control group. In terms of mean weight loss, intervention participants in our study lost on average 5.4 kg (Multimedia Appendix 2).

### Process Evaluation (Adherence)

We examined adherence to the intervention in terms of users versus nonusers among those allocated to use the website (intervention group) (Table 4). “Users: relates to participants who entered information onto the website. “Nonusers” were those participants who never logged on to the website or only logged on to register on the website and did not enter any inputs.

At 3 months, the 9 intervention men who dropped out of the study had all been nonusers of the website from the outset (Figure 2).

**Table 2:** Baseline demographic and anthropometric measures by intervention status.

Outcome measure		Control (n=28)	Intervention (n=33)
**Age (years)**			
	Median (LQ-UQ)^a^	61 (54.5-66.8)	58 (50-67.5)
	Range (min-max)^b^	39 (40-79)	41 (37-78)
White ethnicity, n (%)		28 (100)	33 (100)
**Education (years)**			
	Median (LQ-UQ)	12 (11-12)	12 (10-14)
	Range (min-max)	4 (12-16)	6 (12-18)
**Employment status, n (%)**			
	Employed	11 (39)	9 (27)
	Self-employed	1 (4)	2 (6)
	Unemployed	3 (11)	6 (18)
	Retired	11 (39)	14 (42)
	Caregiver, sick leave, disabled	2 (7)	2 (6)
**Marital status n (%)**			
	Married or in a relationship	25 (89)	24 (73)
	Single	1 (4)	4 (12)
	Divorced or separated	2 (7)	3 (9)
	Widowed	0 (0)	2 (6)
**Weight (kg)**			
	Median (LQ-UQ)	109.3 (96.9-119.0)	106.5 (100.1-115.4)
	Range (min-max)	45.2 (87.2-132.3)	43.2 (86.6-129.8)
**Body mass index (kg/m^2^)**			
	Median (LQ-UQ)	34.4 (31.6-37.0)	33.3 (31.6-36.4)
	Range (min-max)	9.1 (30.3-39.4)	8.2 (30.4-38.6)
**Waist (cm)**			
	Median (LQ-UQ)	119.5 (114-126.8)	118.0 (112-124)
	Range (min-max)	32 (103-135)	33 (100-133)

^a^LQ-UQ: lower quartile to upper quartile.

^b^min-max: minimum to maximum.

**Table 3:** Anthropometric measures across assessment times by group (control vs intervention).

Outcome measure		3 months		12 months	
		Control (n=16)	Intervention (n=24)	Control (n=12)	Intervention (n=20)
**Weight (kg)**					
	Median (LQ-UQ)^a^	105.7 (92.3-114.9)	102.2 (97.4-110.3)	100.7 (91.9-118.1)	99.2 (90.7-106.8)
	Range (min-max)^b^	38.6 (85.5-124.1)	44.3 (84.4-128.7)	40.3 (86.1-126.4)	46.8 (81.2-128)
**Weight change (kg)**					
	Median (LQ-UQ)	–2.2 (–3.7 to –0.9)	–2.35 (–4.5 to –0.9)	–2.5 (–5.0 to 0.2)	–4.3 (–7.8 to –1.0)
	Range (min-max)	9.7 (–8.6 to 1.1)	10.3 (–8.4 to 1.9)	16.1 (–12.6 to 3.5)	21.4 (–18.5 to 2.9)
5% weight loss, n (%)		3 (19)	3 (13)	4 (33)	8 (40)
**BMI^c^ (kg/m^2^)**					
	Median (LQ-UQ)	33.3 (30.8-36.1)	32.2 (31.1-34.5)	33.3 (29.9-36.8)	31.3 (29.8-33.2)
	Range (min-max)	8.7 (28.7-37.4)	9.9 (28.4-38.3)	8.9 (29.3-38.2)	9.9 (27.5-37.4)
**BMI change (kg/m^2^)**					
	Median (LQ-UQ)	–0.7 (–1.1 to –0.2)	–0.9 (–1.4 to –0.2)	–0.8 (–1.6 to 0.8)	–1.7 (–2.7 to –0.3)
	Range (min-max)	3.2 (–3.0 to 0.2)	3.4 (–2.8 to 0.6)	5 (–3.9 to 1.1)	7.8 (–6.5 to 1.3)
**Waist circumference (cm)**					
	Median (LQ-UQ)	118.5 (109.8-124.8)	112.5 (108.5-122.8)	117 (112.3-126.8)	112 (107-121)
	Range (min-max)	22 (106-128)	26 (102-128)	31 (103-134)	21 (103-124)
**Waist circumference change (cm)**					
	Median (LQ-UQ)	–3.0 (–1.3 to –5.0)	–2.0 (–1 to –3)	–2.0 (–3.8 to –1)	–3.5 (–7 to –1.3)
	Range (min-max)	11 (–9 to 2)	14 (–11 to 3)	12.5 (–11 to 2)	19 (–17 to 2)

^a^LQ-UQ: lower quartile to upper quartile.

^b^min-max: minimum to maximum.

^c^BMI: body mass index.

**Table 4:** Website use by intervention participants over the course of the study.

Participants	Baseline (n=33)	3 months (n=33)	12 months (n=33)
Users, n (%)	16 (48)	16 (48)	12 (36)
Nonusers, n (%)	17 (52)	17 (52)	21 (64)

We analyzed website use for those remaining in the intervention group (Table 5). We collected data from 28 men, as 5 intervention participants never registered on the website. Data were examined in relation to their website use between baseline (0 months) and the 3-month data collection point. Data were then examined for website use between 3 months and the end of the study (12 months): 9 intervention participants left the study at the 3-month data collection point and therefore we included 19 men in the analysis. We divided website activity to examine participant’s use in relation to food intake and exercise levels, as well as interactions with allocated dietitians and exercise experts (Table 5). As outlined in Table 1 (intervention description), the proposed number of consultations within the intervention protocol over the first 3 months was 15, with the total number of consultations at the end of the 12-month intervention stated as 27. Table 5 shows that 13 of the 15 intended health professional (dietitian, exercise expert) consultations were delivered at 3 months, whereas at 12 months, 22 out of the 27 were delivered. At the end of the intervention, the number of dietitian-delivered consultations was 1 less than the number scheduled to occur (20 out of 21); however, exercise experts averaged up to 4 fewer than scheduled (2 out of 6). Consultations were delivered fewer times than expected due to nonresponsive participants and time pressures on the health care professionals due to competing demands (eg, full-time jobs). Food-related messages from participants to the health professionals were sent more often than exercise messages. The food intake entries were used more frequently than the exercise entries, with both demonstrating high levels of variability between participants (Table 5). No log-in data were obtained at 3 months, as the website hosts did not record this information, and therefore Table 5 shows only 12-month results. Participants did not use the social support features, interactive chat room, and discussion forums at all. We identified no unintended effects were identified from the pilot trial.

**Table 5:** Website use data averages per intervention participant.

Type of use		0-3 months (n=28)	3-12 months (n=19)
**Dietitian- and exercise expert-sent consultations**			
	Median (LQ-UQ)^a^	13 (12-15)	22 (20-25)
	Range (min-max)^b^	11 (5-16)	15 (14-29)
**Dietitian-sent consultations**			
	Median (LQ-UQ)	12 (10-12)	20 (18-22)
	Range (min-max)	10 (4-14)	10 (13-23)
**Exercise expert-sent consultations**			
	Median (LQ-UQ)	2 (1-2)	2 (1-3)
	Range (min-max)	4 (0-4)	6 (0-6)
**Participant-sent messages**			
	Median (LQ-UQ)	1 (0-8)	9 (0-32)
	Range (min-max)	34 (0-34)	75 (0-75)
**Food-related messages**			
	Median (LQ-UQ)	1 (0-7)	7 (0-29)
	Range (min-max)	33 (0-33)	68 (0-68)
**Exercise-related messages**			
	Median (LQ-UQ)	0 (0-1)	1 (0-2)
	Range (min-max)	7 (0-7)	7 (0-7)
**Food intake entries**			
	Median (LQ-UQ)	8 (1-59)	99 (3-246)
	Range (min-max)	82 (0-82)	330 (0-330)
**Exercise entries**			
	Median (LQ-UQ)	3 (0-26)	22 (2-124)
	Range (min-max)	69 (0-69)	262 (0-262)
**Log-ins**			
	Median (LQ-UQ)	N/A^c^	43 (12-167)
	Range (min-max)	N/A	490 (1-491)

^a^LQ-UQ: lower quartile to upper quartile.

^b^min-max: minimum to maximum.

^c^N/A: not available.

### Process Evaluation (Acceptability)

We conducted 13 semistructured interviews, which enabled us to explore participants’ views in relation to the acceptability of the Web-based weight loss intervention. We classified 7 of the interviewees as active users, participants who logged on to the website and entered information until the end of the study, while 6 of the interviewees were nonusers and therefore were no longer using the website by the end of the study. Interviewees’ ages ranged from 41 to 78 years.

The main themes identified from the interviews were (1) practicality, (2) interaction with the health care professional, and (3) future development of the Web-based intervention.

#### Practicality

This theme was discussed in relation to the participant’s engagement with the intervention.

Rather than have appointments and have to trail wherever it is, I think they’re quicker and they’re more expedient at getting the message across.

Participant 20, age 58, active user

It’s flexible, communication hasn’t got to be restricted to clinic type hours…you can do it when they want…and you haven’t got issues with them cancelling.

Participant 17, age 57, active user

The website was viewed as more accessible than conventional treatment, such as face-to-face meetings, overcoming the difficulty of fitting restricted clinic hours and appointment times into everyday life. Participants also referred to the website as “easy to use” once they had become accustomed to it.

#### Interaction With the Health Care Professional

Interaction between the health care professionals and the participants was referred to with regard to the relationship built up during the course of the study.

However, participants deemed having a one-off initial face-to-face meeting with the dietitian as important for their future Web-based interaction.

This is who I’m talking to and you’re not just I don’t know a nameless blob out there somewhere they know you’re a real person.

Participant 4, age 58, nonuser

That would've helped me because it would have felt more like I knew who was watching. It felt a lot more distant with the exercise person. So it felt like it could’ve been that that message has gone across the board.

Participant 60, age 53, nonuser

There was no face-to-face meeting with the exercise experts, and many participants believed that having the opportunity to talk through expectations would possibly have avoided misconceptions that appeared to take place during the study; that is, that the ability level of the participants could have been witnessed and assessed by the exercise expert, and that the exercise experts would have been able to vocalize from the beginning that slow and gradual increase of physical activity was recommended, whereas participants often assumed that they were expected to do vigorous exercise and this was not suitable for them.

The interaction between the health care professionals and the participants was also referred to in relation to monitoring and receiving guidance on their progress.

Acknowledgment of the participants’ self-monitoring appeared to be an essential aspect in the provision of guidance to ensure they knew it was a human and personal response rather than simply an automated message.

But it obviously is getting monitored and that gives me the confidence to carry on using it. It’s a trust thing as much as anything else, and I’m more than trustful of it.

Participant 17, age 57, active user

Feedback consultations seemed to provide participants with the reassurance that they could be helped with any issues, comforted by the knowledge that professional guidance was available if needed.

#### Future Development of the Web-Based Intervention

Aspects that could be developed in future emerged through participants highlighting technical or practical issues and with participants suggesting potential changes that could be made to improve the Web-based intervention.

A suggested improvement of the website was for the food database to be in alphabetical order to save participants time.

I think the menus require some pretty solid attention. Obviously, they are trying to be helpful, but it is the way they are presented. It is labour-intensive. [Participant 50, age 78, nonuser]

Participants also suggested that including healthy eating or exercise recommendations would allow them to compare their own performance against these set recommendations. Other comments emerged during the interviews with regard to the website needing more color to be more appealing.

Another observation I would make is that on the website, there is an awful lot of text. There are not many cheerful graphics.

Participant 45, age 62, active user

This suggests that, as well as functionality and professionalism, the website also needs to be visually attractive to make it interesting to users.

Suggestions were raised on ways to improve the website in order to aid productivity and ease of use for both health professionals and participants, with the overall objective being to create a website that could be less time consuming and more straightforward for users to operate while providing adequate support. Examples of these suggestions are organizing the food database alphabetically to make finding a meal or food choice quicker, adding the ability to enter free-text calorie information, making the website more colorful and appealing, adding healthy eating or exercise recommendations to compare against their own progress, and adding the ability to view a full week of food or exercise inputs rather than just daily reports.

### Sample Size Calculations for a Main Trial

We calculated sample size, as issue 1 in Table 6 reports [44], using PS: Power and Sample Size Calculation version 3.1.2 [45] to identify the sample size needed for a main trial, while we based our other calculations on response and retention rates from this pilot study. Sample size was calculated based on the main trial being a superiority trial and for a target difference of 5% between the control and the intervention arms in terms of percentage weight change from participants’ initial baseline weight to follow-up at 12 months (eg, 5% loss in the intervention group versus 0% in the control group, or 6% vs 1%), with 90% power, 5% significance level, one-to-one allocation, and analysis with independent-samples *t* test; a standard deviation of weight loss change of 5.6% was assumed.

**Table 6:** Summary of findings against 14 methodological issues for feasibility research^a^.

Methodological issues		Findings	Evidence	Suggested improvements for a full trial
1.	Did the feasibility/pilot study allow a sample size calculation for the main trial?	Measure of variability and retention rates was identified. Sample size for main trial was calculated.	Target of 60 was achieved.	Sample sizes were calculated to inform main trial requirements. Number of practices required: 12 (based on average of 138 eligible patients identified per practice). Number of participants needing to be identified and contacted: 1587 (based on consent rate of 6.3%). Number needing to be randomly allocated: 100 (based on retention rate of 52%). Number needed at 12 months to detect target difference: 54 (27 per arm).
2.	What factors influenced eligibility and what proportion of those approached were eligible?	High numbers of eligible men (968) were identified from GP^b^database searches.	5 out of 61 approached were ineligible.	Inaccurate body mass index records in GP databases led to 5 ineligible participants, a small number but an issue to consider when contacting general practices.
3.	Was recruitment successful?	Recruitment was successful.	Target of 60 was achieved. Response to study invitation was 9% of identified men.	Recruitment via GP database searches was effective. Invitation letters could be revised, to be based on behavior change principles, to potentially increase response rate and those recruited.
4.	Did eligible participants consent?	Conversion to consent was high.	Of the 61 eligible men, all were recruited.	The consent process was successful and could stay the same for a main trial.
5.	Were participants successfully randomized and did randomization yield equality in groups?	Randomization worked well.	Allocation was concealed. Groups were of fairly equal size and were well balanced on stratification variables.	Randomization and stratification worked well and could progress into a main trial.
6.	Were blinding procedures adequate?	Blinding was not possible and was not planned.	Blinding was not implemented.	Blinding would not be possible in a main trial.
7.	Did participants adhere to the intervention?	Fewer than half of the participants adhered to the intervention website.	16 out of 33 (48%) allocated intervention participants actively used the intervention website, with 12 out of 33 (36%) still engaged at 12 months.	The use of incentives could aid both adherence and retention. Improvements to the website, suggested in the process evaluation, were mentioned in relation to increasing adherence.
8.	Was the intervention acceptable to the participants?	The intervention appeared to be acceptable to participants.	All eligible participants consented once full study information was explained. The majority of participants interviewed believed the intervention to be feasible to implement within the UK National Health Service.	A Web-based weight loss intervention was identified as acceptable.
9.	Was it possible to calculate intervention costs and duration?	These were not assessed within this pilot trial.	No costs were calculated.	Cost analysis would be conducted in a main trial to assess the cost effectiveness of the intervention.
10.	Were outcome assessments completed?	Anthropometric measures were completed well.	Anthropometric measures were completed by all participants remaining in the study.	Face-to-face anthropometric measures could be used in a future trial.
11.	Were outcomes measured those that were the most appropriate outcomes?	Outcome measures used did assess main areas of interest.	Anthropometric measures allowed health outcomes to be measured.	Outcome measures would be suitable to measure in a full trial.
12.	Was retention to the study good?	Attrition was substantial.	Remaining men: 3 months: 73% intervention, 57% control 12 months: 61% intervention, 45% control.	Incentives could be used, as in previous research, to aid both adherence and retention.
13.	Were the logistics of running a multicenter trial assessed?	Logistics for running a multicenter trial identified no problems during the trial.	However, the number of participants recruited from each general practice was largely influenced by the number of eligible participants identified in the GP database search; 50 of the 61 (82%) recruited were from the 3 practices where the greatest number of eligible participants were identified.	The logistics for running a multicenter trial were effective and could be used in a main trial. Focusing on larger practices may be most effective.
14.	Did all components of the protocol work together?	Components had strong synergy.	No difficulties were identified in the ability to implement any of the study processes. Participants were recruited, were randomly allocated, and progressed into the appropriate trial arm smoothly.	The protocol allowed all components to work well together.

^a^Based on Shanyinde et al [44].

^b^GP: general practitioner.

### Methodological Issues and Possible Solutions for a Main Trial

The CONSORT guidelines recommend that pilot trials provide prespecified criteria to judge whether to proceed with a future trial [39]. A previous study identified 14 methodological issues for feasibility research to consider when making the decision to proceed with a future trial [44]. Therefore, Table 6 outlines this study’s findings, evidence, and suggested improvements for a main trial. Findings detailed in Table 6, along with the overall findings of the study, have enabled the recommendation that a main trial of the intervention should not proceed without the modifications and improvements identified. The main challenges that arose within this study were the low response rates to express interest in joining the study and low retention rates. Table 6 outlines possible solutions to these problems. A potential solution to the low response rates to join the study is the use of incentives for participation to try to increase uptake and recruitment. However, we achieved the recruitment target of 60 participants; therefore, although a low number responded to the GP invitation letters, we were also able to reach a large audience. In relation to the challenge of retaining participants in the study, the interview findings identified that improvements to the website would have encouraged participants to use the website more frequently and would have upheld their interest to a greater degree. Participants viewed the website as acceptable. The conversion to consent, recruitment, and randomization protocols were all found to be effective.

## Discussion

### Principal Findings

We achieved the recruitment target. Participant interviews identified the Web-based intervention as an acceptable method of delivery for weight loss; however, improvements to the website were suggested in relation to ease of use and to maintain adherence. Participants’ Web-based messages to health professionals tended to be directed to the dietitians rather than the exercise experts. However, it was also evident that the dietitians achieved more of their scheduled number of professional-initiated consultations than the exercise experts. Data completion rates at each time point were sufficient to inform sample size calculations for a future definitive trial.

### Comparison With Prior Work

To address problems identified in pilot studies, solutions should relate to study context, trial design, the intervention, or all 3 of these, and whether these could be effective or feasible within trial or real-world settings [46]. This study has examined these feasibility issues noted in Table 6.

In agreement with this study, findings from previous research [47] identified Web-based weight loss interventions as acceptable and feasible, including Brandt et al [37], the Danish study that originated the My Dietitian website.

We used general practices in this study, as this is how patients would be referred to exercise experts or dietitians for weight loss within the NHS. Within this pilot trial, only 9% of those invited expressed an interest in the study, but we met the recruitment target of 60 participants. A previous study that contacted patients via GP mailouts achieved a 6.5% response rate [48]. A suggestion to improve research study recruitment strategies, such as GP mailouts, is the use of opt-out techniques. Although these are disliked by ethics committees, previous research suggested it can increase response rates by 12% and should be used in low-risk groups, as opt-in techniques can result in a biased sample [49].

Previous research has shown great variability in Web-based weight loss trial recruitment levels, ranging from 6% to 83%, and in terms of the recruitment techniques implemented. Previous methods for recruitment range from a wider audience approach, such as advertisement techniques [50-53], to a targeted approach through GP mailouts or referrals [47,54], with varied success. Many of the studies used several methods of recruitment rather than one single approach [50,51,55,56]. One difficultly when comparing against previous research is the reporting of response and recruitment numbers. Published work can often report on the number screened for eligibility assessment and not the actual numbers who viewed the invitation, with the reach of some recruitment strategies, such as advertisements, unknown [37,47].

Attrition levels from previous studies range from 17.4% to 51.4% for Web-based intervention arms and 15.2% to 35.5% for control arms [47,53,57] at 12 months. This study experienced higher rates of attrition for the control arm but rates similar to those in previous research for the intervention arm. However, the control group had higher attrition than in previous research. Control groups have been discussed by Morgan et al [58], with the suggestion that a minimal intervention is necessary due to some form of intervention being more acceptable to participants than no intervention, which therefore prevents attrition, with attrition rates identified as 29% by 12 months. Tate et al [47] had similar attrition rates for both included groups—an Internet-only group and an e-counselling group—with an overall attrition of 16%. Their study used incentives for appointment attendance, which could be a potential improvement for our study. Our study used usual care, which we discovered to be near nonexistent in terms of specific weight loss treatment, with only 1 participant being referred to exercise classes. For this study, it was deemed important, and was achieved, to identify what usual care constituted for this population within the NHS. However, an improvement may be the use of a minimal intervention as the control group. Further investigation into different modes of delivery would also be beneficial to identify whether delivering an intervention in person or via the Internet would affect the overall findings.

In terms of mean weight loss, intervention participants in our study lost on average 5.4 kg, which is greater than in previous studies, which had losses of 4.4 kg [47], 4.6 kg [54], and 5.3 kg [58], although lower than Brandt and colleagues’ study, in which mean weight loss was 7 kg [37].

### Strengths and Limitations

In addition to the pilot RCT, it was possible to conduct a process evaluation alongside the trial, which enabled us to investigate participants’ views and to track website use. We attributed the nondelivery of over half of the exercise consultations to the change in job status of the exercise experts, as well as nonresponsive participants. However, these reasons emerged from the process evaluation interviews. The health care professionals were not required to provide reasons for nondelivered consultation, and this was not recorded in the website use data. Therefore, we do know how many consultations were not delivered due to nonresponsive participants and how many were due to the health care professionals. A potential improvement going forward would be to require the health care professionals to enter reasons for missed consultations. As the health care professionals did not meet the proposed number of consultations, we suggest that large-scale trials should employ health care professionals as research study staff. If this is not possible, at least providing health care professionals with dedicated time to work on the study would be beneficial to the fidelity of intervention delivery rather than having their involvement with the study being in addition to their other full-time employment.

Participants were sent details of their username and operational directions in a postal letter in advance of the face-to-face meeting. In hindsight, the face-to-face meeting could have been a potential opportunity to explain the functions and resources within the website and explore how to effectively interact with it. This identifies a potential improvement and refinement of the intervention procedures for future research.

The intervention is reliant on feedback from a health care professional and, unless provided through the NHS, this would not be possible in a standard weight loss website. However, this study demonstrates how a Web-based weight loss intervention may be used for a high-risk population. The study sample captured a wide age range of 41 to 79 years and contained a range of employment status (unemployed, employed, retired). A limitation of the study sample is the lack of ethnic diversity: the sample was all white British men. South Asian and Black African groups are known to be twice as likely to develop type 2 diabetes and therefore a future study should aim to recruit ethnic minority groups to identify whether a Web-based intervention is acceptable to people of different ethnicities.

It is important to acknowledge the conflict between conducting a rigorous RCT and the need to keep up with the fast progression of technologies. Evaluation research faces the reality of falling behind commercial companies with the ability to regularly update their websites or apps. Large companies may have the advantage of greater financial stability and flexibility of funding and resources in contrast to academic research, where budgets can be extremely constricted and individual costs and resources tend to be outlined in advance of receiving funding. However, RCT methodology remains the most robust way of determining the effectiveness of an intervention [59]. One way to keep up-to-date with technology and maybe another potential improvement for the study is the use of a mobile phone app, in replacement of or in addition to a computer-based website. Mobile phones have now overtaken laptops or desktops as the devices used to access the Internet [60]. Although the study website was accessible via a mobile phone, the creation of an app may make the format easier to access on a mobile phone and potentially improve engagement and adherence. Over 12 months, each participant had 3 visits by the researcher at either their home or general practice, with data collection typically ranging between 20 minutes and 1 hour per visit. This level of face-to-face assessment with participants could be feasible in a main trial. However, the use of electronic scales to measure and transmit weight status to the research team, as implemented within the NULevel study [61], may be a more efficient and feasible method of data collection in a definitive trial.

### Implications for Policy, Practice, and Further Research

Research is lacking with regard to implementing a Web-based weight loss intervention within the NHS. Given the high number of obese patients and NHS resources being increasingly stretched, services are struggling to provide sufficient referrals. Therefore, alternative modes of delivery, with the potential to reduce health professional input and time per patient while still enabling individual and tailored care, need to be investigated to identify if they can be effective and thus benefit the NHS. Although not powered to assess changes in outcomes, the descriptive statistics show positive indications of increased weight loss (in kilograms and as a percentage), reduced waist circumference, and decreased BMI for the intervention group from 3 to 12 months, in comparison with the control group. This research provides preliminary findings that recruitment of men with type 2 diabetes is possible within a Web-based intervention. Suggested improvements to the website were valuably gained from the parallel process evaluation and could be incorporated to potentially improve adherence and retention in future research.

## Appendix

Multimedia Appendix 1 CONSORT-EHEALTH checklist V1.6.1.

Multimedia Appendix 2 Anthropometric measures mean (SD).

## Abbreviations

BMI: body mass index
CALO-RE: Coventry, Aberdeen, and London-Refined
CONSORT: Consolidated Standards of Reporting Trials
GP: general practitioner
NHS: National Health Service
RCT: randomized controlled trial
