# Supplementary material for: Web-Based Weight Loss Intervention for Men With Type 2 Diabetes: Pilot Randomized Controlled Trial
Source: JMIR Diabetes. 2017 Jul 7;2(2):e14. doi: 10.2196/diabetes.7430 (PMC6238853; doi:10.2196/diabetes.7430)
Supplement: Multimedia Appendix 2 [file diabetes_v2i2e14_app2.pdf]

	**Baseline**		**3 months**		**12 months**	
	**Control (n=28)**	**Intervention (n=33)**	**Control (n=16)**	**Intervention (n=24)**	**Control (n=12)**	**Intervention (n=20)**
**Outcome measure**	Mean (SD)	Mean (SD)	Mean (SD)	Mean (SD)	Mean (SD)	Mean (SD)
**Height (cm)**	176.9 (6.9)	177.7 (7.3)	177.0 (6.7)	177.7 (7.7)	176.0 (5.7)	178.2 (8.4)
**Weight (kg)**	108.5 (13.4)	107.2 (10.9)	104.4 (12.2)	103.6 (10.2)	103.8 (14.3)	100.7 (12.3)
**Weight change (kg)**	-	-	-2.6 (2.7)	-2.6 (2.7)	-2.8 (4.4)	-5.4 (5.9)
**5% weight loss (No. (%))**	-	-	3 (18.8)	3 (12.5)	4 (33.3)	8 (40)
**BMI**	34.6 (3.0)	33.9 (2.6)	33.3 (2.8)	32.8 (2.4)	33.4 (3.3)	31.6 (2.6)
**BMI change**	-	-	-0.9 (0.9)	-0.8 (0.9)	-0.9 (1.4)	-1.3 (2.0)
**Waist circumference (cm)**	119.7 (8.9)	117.7 (7.6)	117.7 (7.9)	115.4 (7.6)	118.2 (9.3)	113.1 (7.1)
**Waist change (cm)**	-	-	-3.2 (2.7)	-2.5 (3.1)	-2.6 (3.5)	-4.5 (4.9)
